# Paediatric magnetoencephalography and its role in neurodevelopmental disorders

**DOI:** 10.1093/bjr/tqae123

**Published:** 2024-07-08

**Authors:** Natalie Rhodes, Julie Sato, Kristina Safar, Kaela Amorim, Margot J Taylor, Matthew J Brookes

**Affiliations:** Sir Peter Mansfield Imaging Centre, School of Physics and Astronomy, University of Nottingham, University Park, Nottingham NG7 2QX, United Kingdom; Program in Neurosciences & Mental Health, Hospital for Sick Children, Toronto, ON M5G 0A4, Canada; Department of Diagnostic & Interventional Radiology, Hospital for Sick Children, Toronto, ON M5G 0A4, Canada; Program in Neurosciences & Mental Health, Hospital for Sick Children, Toronto, ON M5G 0A4, Canada; Department of Diagnostic & Interventional Radiology, Hospital for Sick Children, Toronto, ON M5G 0A4, Canada; Program in Neurosciences & Mental Health, Hospital for Sick Children, Toronto, ON M5G 0A4, Canada; Department of Diagnostic & Interventional Radiology, Hospital for Sick Children, Toronto, ON M5G 0A4, Canada; Program in Neurosciences & Mental Health, Hospital for Sick Children, Toronto, ON M5G 0A4, Canada; Program in Neurosciences & Mental Health, Hospital for Sick Children, Toronto, ON M5G 0A4, Canada; Department of Diagnostic & Interventional Radiology, Hospital for Sick Children, Toronto, ON M5G 0A4, Canada; Department of Psychology, University of Toronto, Toronto, ON M5S 2E5, Canada; Department of Medical Imaging, University of Toronto, Toronto, ON M5T 1W7, Canada; Sir Peter Mansfield Imaging Centre, School of Physics and Astronomy, University of Nottingham, University Park, Nottingham NG7 2QX, United Kingdom; Cerca Magnetics Limited, Nottingham NG7 1LD, United Kingdom

**Keywords:** MEG, magnetoencephalography, neurodevelopment, ASD, ADHD

## Abstract

Magnetoencephalography (MEG) is a non-invasive neuroimaging technique that assesses neurophysiology through the detection of the magnetic fields generated by neural currents. In this way, it is sensitive to brain activity, both in individual regions and brain-wide networks. Conventional MEG systems employ an array of sensors that must be cryogenically cooled to low temperature, in a rigid one-size-fits-all helmet. Systems are typically designed to fit adults and are therefore challenging to use for paediatric measurements. Despite this, MEG has been employed successfully in research to investigate neurodevelopmental disorders, and clinically for presurgical planning for paediatric epilepsy. Here, we review the applications of MEG in children, specifically focussing on autism spectrum disorder and attention-deficit hyperactivity disorder. Our review demonstrates the significance of MEG in furthering our understanding of these neurodevelopmental disorders, while also highlighting the limitations of current instrumentation. We also consider the future of paediatric MEG, with a focus on newly developed instrumentation based on optically pumped magnetometers (OPM-MEG). We provide a brief overview of the development of OPM-MEG systems, and how this new technology might enable investigation of brain function in very young children and infants.

## Introduction

### What is MEG?

Magnetoencephalography (MEG) allows non-invasive imaging of neurophysiological activity, via the measurement of magnetic fields at the scalp surface, generated by neuronal current flows. It is the magnetic counterpart of electroencephalography (EEG), the most common clinical tool for assessing brain function, which measures changing electrical potential across the scalp surface caused by the same neural currents. Because they provide ‘direct’ (i.e., electrical) measurement of brain function, MEG and EEG are advantageous compared to techniques like functional magnetic resonance imaging (fMRI) and near infra-red spectroscopy, which give only ‘indirect’ assessment based on haemodynamics as a proxy of brain function. Unfortunately, EEG is limited by sensitivity to artefacts, and electrical potentials being spatially distorted by the high electrical resistance of the skull, which leads to poor spatial resolution. In contrast, magnetic fields are less susceptible to artefact and pass through the skull relatively undisturbed, simplifying source modelling and allowing signals to be traced back to where they originated, with accuracy of a few millimetres^1^. Because of this, MEG can examine (with millisecond temporal and millimetre spatial accuracy) the spatio-temporal dynamics of brain network activity, providing a window into brain function in health and disease.

### What does MEG measure?

**Figure 1.**
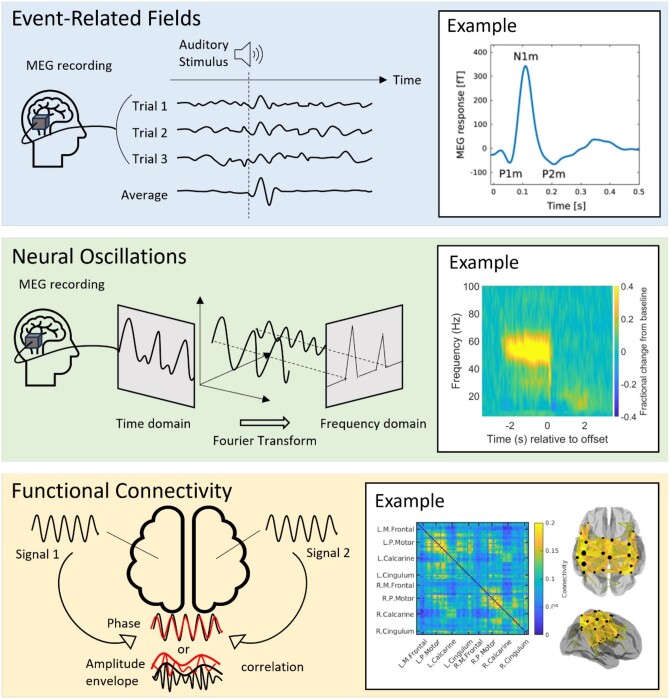
Types of MEG signals. The top-panel shows event-related fields: the schematic shows how time and phase locked signal fluctuations can be averaged over trials to create an evoked response. The example on the right shows a typical response to an auditory tone. From Hajizadeh A, Matysiak A, May PJC, König R. Explaining event-related fields by a mechanistic model encapsulating the anatomical structure of auditory cortex. Biol Cybern. 2019;113(3):321-345. doi: 10.1007/s00422-019-00795-9; http://creativecommons.org/licenses/by/4.0/)^13^. The middle panel shows a neural oscillatory modulation. In the schematic, we show how a single oscillatory waveform can be decomposed into its constituent frequency bands. The example on the right from Hill RM, Devasagayam J, Holmes N, et al. Using OPM-MEG in contrasting magnetic environments. Neuroimage. 2022;253:119084. doi: 10.1016/j.neuroimage.2022.119084; http://creativecommons.org/licenses/by/4.0/^14^ shows a time-frequency spectrogram with modulation of gamma (30+ Hz) activity during a task where subject watch a visual stimulus. Blue reflects a decrease and yellow reflects an increase in amplitude relative to the baseline (here baseline is defined as −3.4 to −2.5 seconds). The bottom panel shows functional connectivity. The schematic shows how it is possible to derive waveforms from spatially separate brain regions and look for a relationship between them. The example on the right from Boto E, Hill RM, Rea M, et al. Measuring functional connectivity with wearable MEG. Neuroimage. 2021;230:117815. doi: 10.1016/j.neuroimage.2021.117815; https://creativecommons.org/licenses/by/4.0/^15^ shows a matrix representing connectivity between a set of parcellated brain regions. The ‘glass brain’ shows the spatial signature of the strongest 200 connections. In this example, we show beta band connectivity, which is strongest in the sensorimotor system^15^. Abbreviation: MEG = magnetoencephalography.

MEG is typically used to measure 3 types of neuronal signatures:

- **Event-related fields**: time-locked evoked responses to repeated events, such as sensory stimulation or cognitive demand. Upon stimulus presentation, there is a time- and phase-locked signal fluctuation which becomes clearly observable (over ongoing activity) when data are averaged over many trials (see Figure 1, upper panel).
- **Neural oscillations:** rhythmic patterns of activity that underlie a range of cognitive processes. In adults, these signals are typically separated into canonical frequency bands: delta (0.1-3 Hz), theta (4-7 Hz), alpha (8-13 Hz), beta (13-29 Hz) and gamma (30+ Hz); however, these ranges vary in infants and children^2^. These oscillations are present even in the absence of a task and are modulated by task performance (see Figure 1, centre panel).
- **Functional connectivity:** the synchronicity of neural oscillations measured in different brain regions, providing insight into brain networks. This can be computed via a range of metrics, including amplitude envelope correlation (when the amplitudes of oscillations measured in 2 or more brain areas co-fluctuate, implying a network connection) or phase synchrony (when there is a fixed phase relationship between signals from 2 or more regions, again implying a network connection) (see Figure 1, lower panel).

### How is MEG used?

Clinically, MEG is approved for applications relating to epilepsy^3^. While antiepileptic drugs are the primary treatment for epilepsy, drug-resistant forms may be treated by surgery, where the affected area is resected or disconnected. Presurgical assessment is carried out to identify the epileptogenic zone^3–6^ and abnormal interictal epileptiform discharges (IEDs) have been shown to have high spatial concordance^4^. Thus, localizing the cortical origin of IEDs with MEG can provide a valuable indication of the location of the epileptogenic cortex. While in principle EEG can also do this, MEG benefits from greater spatial accuracy^7^, similar to invasive recordings^8^^,^^9^ (see also^7^). Secondly, MEG can also be used to map the eloquent cortex surrounding the epileptogenic zone, and this can minimize the risk of neurological deficits post-surgery^5^^,^^6^. In this application, MEG is used alongside other neuroimaging techniques, including EEG, stereoEEG, and MRI^9^. Critically, recent work in 1000 epilepsy cases showed that those in whom MEG-identified regions were resected were significantly more likely to achieve seizure freedom^10^. Additionally, outcomes are improved when surgical treatment is administered as early as possible^4^^,^^11^. Given this evidence and the high prevalence of epilepsy in children, paediatric MEG represents an important clinical tool (for a review of the use of MEG in epilepsy, see Otsubo et al^12^).

Outside its clinical use, MEG has been a valuable research tool for more than 5 decades, with over 16,000 research documents mentioning the technique since 1977 (Scopus search ‘magnetoencephalography’). Studies have employed MEG to investigate brain function across the lifespan—from neurodevelopment to neurodegeneration. Much of what we know about brain function—in particular electrophysiology—has either been derived from or supported by MEG studies. In this review, we describe the application of MEG in 2 neurodevelopmental disorders. We have chosen not to focus on epilepsy (which is well served by other reviews^12^), but rather focus on autism spectrum disorder (ASD) and attention-deficit hyperactivity disorder (ADHD), where MEG is having a significant impact on our understanding of the neuropathophysiology that underlies symptomology. While most of the existing MEG paediatric literature has focused on ASD, we have chosen to highlight both neurodevelopmental disorders given the high rates of comorbidity; recent reports suggest that 50%-70% of individuals with ASD also have a diagnosis of ADHD^16^. Although the MEG studies in ADHD are more limited, we believe they provide important insights into the neurophysiological underpinnings of the disorder.

In what follows, we first describe the development of MEG hardware and its use in paediatric populations. We then review the application of this hardware to ADHD and ASD. Finally, we explore the evolving landscape of MEG technology—particularly the promise of optically pumped magnetometers (OPMs) to detect MEG signals. We discuss the relevance of such systems for paediatric neuroscience, and their potential impact on diagnosing and understanding neurodevelopmental disorders in children from early infancy.

## MEG hardware

### From sensor to system

MEG was introduced in 1968, when Canadian physicist David Cohen measured the magnetic field generated by neural oscillations in the alpha band (which had first been observed by Hans Berger using EEG in 1929^17^). Initially, the signal was detected using a single induction coil^18^; however, this was later replaced by a more sensitive instrument known as a superconducting quantum interference device (SQUID), with the first MEG measurements (using a single SQUID) carried out in 1972^19^. Systems slowly grew in size, with multiple SQUIDs enabling simultaneous measurement of the magnetic field at multiple locations around the head (known as multiple channels). By the late 20th century, multi-channel systems providing whole-head coverage had been constructed, and nowadays typical MEG systems have up to 300 channels and offer good coverage of most of the cortex (in adults).

Systems are complex: SQUID-based sensors require cryogenic cooling within a liquid helium dewar to maintain the superconducting state that enables them to work (see Fagaly^20^ for a comprehensive review). Consequently, MEG systems are formed from an array of sensors housed in a one-size-fits-all helmet that maintains a thermally insulating gap between the scalp surface and the (extremely cold) sensors. Cryogenic infrastructure is therefore a critical requirement, and most modern systems also need helium recyclers; these reliquefy helium as it boils off inside the MEG system. This reduces the requirement for refilling the system with liquid helium, which requires significant training, is costly, and is bad for the environment.

The magnetic fields generated by the brain are small (∼10-1000 fT)—around a billion times smaller than the Earth’s magnetic field and much smaller than fields generated by electronic equipment or mains electricity supplies. MEG systems are therefore housed inside magnetically shielded rooms with walls consisting of multiple layers of high permeability and conductivity metal, to reduce low- and high-frequency magnetic interference, respectively. Sensor arrays must also be designed to reduce interference—typically via the measurement of magnetic field gradients, which helps to reduce the effect of distal sources of magnetic field^21^. Complete systems also require a means to comfortably seat a participant with their head close to the array, deliver interactive stimulation to the participant (e.g., via a screen and a set of buttons for the patient to press) and monitor the participant (e.g., via video)—this is particularly important for patients. Finally, systems need a means to not only control the SQUID array but also extract, collate, and process the rich data that are generated. In sum, conventional MEG systems are complex and expensive machines; nevertheless, they represent an extremely versatile means to generate high-fidelity electrophysiological data.

### Paediatric MEG systems

Conventional MEG systems are typically designed around the adult head, meaning children with smaller head sizes have a greater separation between the MEG sensors and the generators of the signal (neural assemblies in the brain). Due to an inverse square relationship between magnetic field magnitude and distance from the source, this results in a substantially lower signal-to-noise ratio (SNR) when scanning young children^22^. In addition, with smaller heads, there is more space in the MEG helmet, allowing for increased head movements which further degrades data quality^23^.

Despite these limitations, most paediatric MEG studies have been conducted using adult-sized MEG systems^24^. While possible, these studies must address the confound that—on average—younger children will have smaller heads and therefore lower SNR. Consequently, there are far fewer MEG studies with very young children (<5 years) and most studies concentrate on school-age children—while extremely valuable, these studies miss the most dramatic neurodevelopmental changes that occur in the first few years of life. To scan infants (i.e., under 1 year old) using an adult MEG typically involves separate measurements for each hemisphere, with participants first laid on one side^25^, and then the other, to attain whole-brain coverage over 2 independent experiments. Such studies typically have a very low data retention rate, often rejecting more than half of the recruited infant participants due to unsuccessful scans or insufficient data quality^26^^,^^27^. In addition, they preclude measurement of brain-wide networks where one requires simultaneous data from all brain regions.

**Figure 2.**
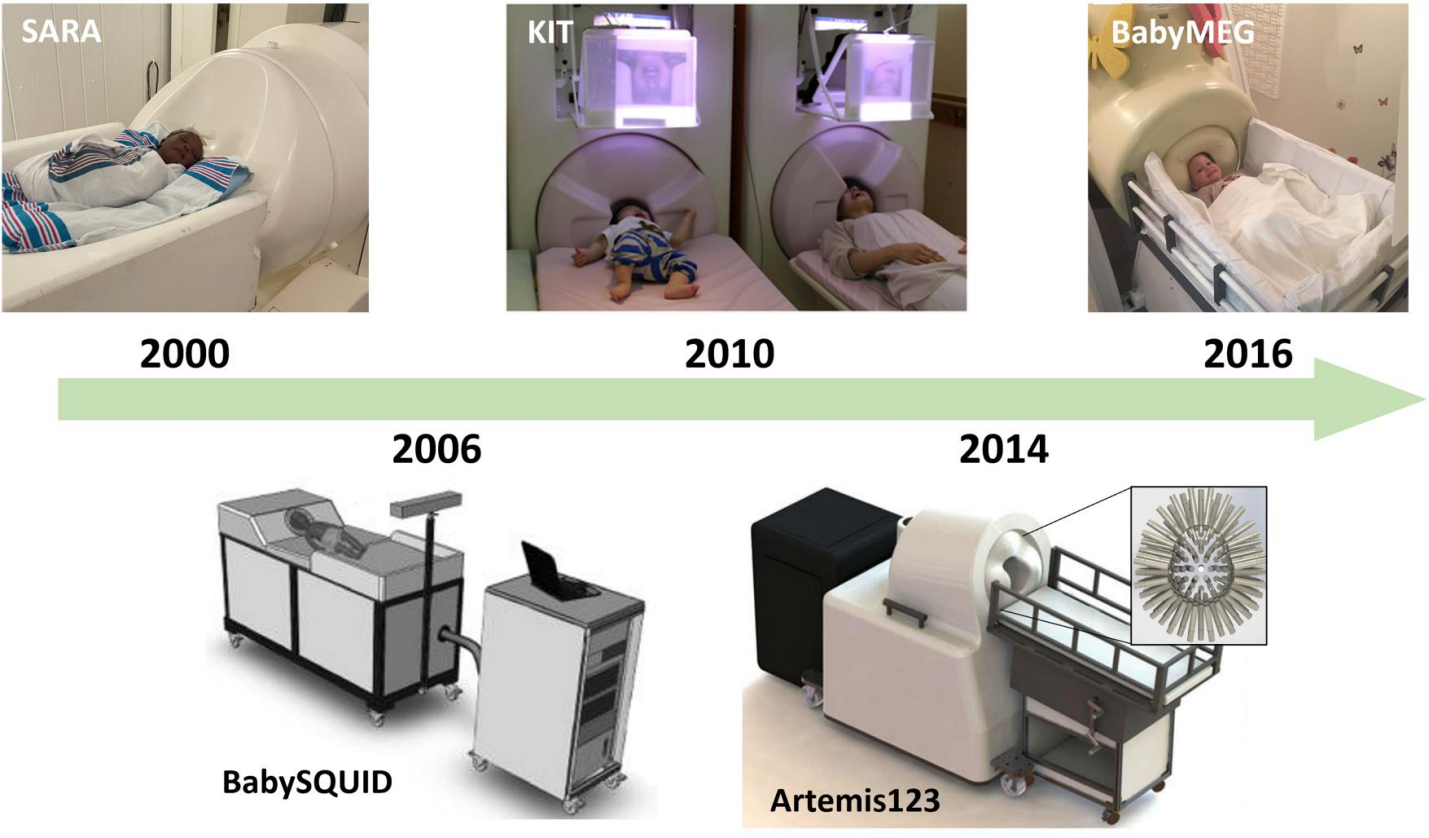
MEG paediatric systems. By date, the SARA system with an infant participant (Image provided by SARA Lab at University of Arkansas Medical Sciences); a schematic of the BabySQUID system (reprinted from Okada Y, Pratt K, Atwood C, et al. BabySQUID: a mobile, high-resolution multichannel magnetoencephalography system for neonatal brain assessment. Rev Sci Instrum. 2006;77:024301,^29^ with the permission of AIP Publishing); the KIT infant system (left) next to the adult system (Hirata M, Ikeda T, Kikuchi M, et al. Hyperscanning MEG for understanding mother-child cerebral interactions. Front Hum Neurosci. 2014;8:118. doi: 10.3389/fnhum.2014.00118; https://creativecommons.org/licenses/by/3.0^35^); the Artemis123 (Roberts TPL, Paulson DN, Hirschkoff E, et al. Artemis 123: development of a whole-head infant and young child MEG system. Front Hum Neurosci. 2014;8:99; doi: 10.3389/fnhum.2014.00099; https://creativecommons.org/licenses/by/3.0/^33^) and the BabyMEG (top right; reprinted from Okada Y, Hämäläinen M, Pratt K, et al. BabyMEG: a whole-head pediatric magnetoencephalography system for human brain development research. Rev Sci Instrum. 2016;87(9):094301^34^ with the permission of AIP Publishing). Abbreviations: MEG = magnetoencephalography; SARA = SQUID Array for Reproductive Assessment.

There have been several attempts to address this instrumentation gap, with the introduction of child- or infant-sized MEG systems designed around smaller head sizes (see Figure 2). This reduces the source-to-sensor distance and therefore increases signal sensitivity, while still using the same (cryogenic) sensing technology. The first system was the ‘SQUID Array for Reproductive Assessment’, a device designed for foetal, neonatal, and infant recordings. The device used 151 SQUID-based gradiometers in a concave array to conform to the pregnant abdomen or to an infant’s head using an attached cradle^28^. Due to the wide array design, infant recordings still required 3 separate sessions with the baby laid down in 3 orientations to gain whole-head coverage. This system was followed by the BabySQUID which housed 76 first-order gradiometers in an ellipsoid-shaped helmet designed to cover approximately half the head^29^ (again precluding whole-brain coverage). The first whole-head paediatric system became available in 2010, with the development of the KIT system^30–32^, initially comprising 64 channels (later upgraded to 151 channels) and designed to fit >90% of 5-year-old children. For younger infants, the Artemis 123 was designed to be compatible from birth to 3 years old (designed around the median 3-year-old head in the United States)^33^. The most recent SQUID-based infant system is the BabyMEG, which has a dense array of 375 magnetometers in a helmet designed to fit up to 4 years of age^34^. These systems all help to address the problem of small heads. However, for neurodevelopmental studies where the aim is to scan large numbers of individuals at different ages and look for changes in brain function, there remains a significant confound: younger participants have to be scanned in a different system to older children or adults, and even with paediatric systems, a newborn baby’s head remains much smaller than that of a 4-year-old, meaning the same limitations of changing SNR apply.

## Paediatric MEG in ASD and ADHD

Despite its limitations, MEG has been instrumental in mapping the neural mechanisms underlying social and cognitive processes, which undergo protracted maturation over childhood and adolescence. These findings can also help us better understand altered brain development and sequelae in neurodevelopmental disorders. ASD and ADHD are the 2 most prevalent neurodevelopmental disorders, with global rates of 1.5% and 5% in children, respectively^36^^,^^37^. ASD is marked by clinically significant difficulties in language, communication, and social interactions, while ADHD is characterized by persistent symptoms of inattention, hyperactivity, and impulsivity^38^; although not a central feature of the disorder, social impairments are also present in ADHD^39^.

The symptomology of these disorders has meant that much of the MEG research has focused on auditory processing (hypothesized to underlie poor verbal or attentional abilities), emotional face processing (thought to underlie poor social skills), or task-free resting-state activity (thought to reflect intrinsic brain function), which will be covered in the following sections. Due to space limitations, other MEG studies investigating attention, language, and sensory processing will not be covered.

### Auditory processing

**Figure 3.**
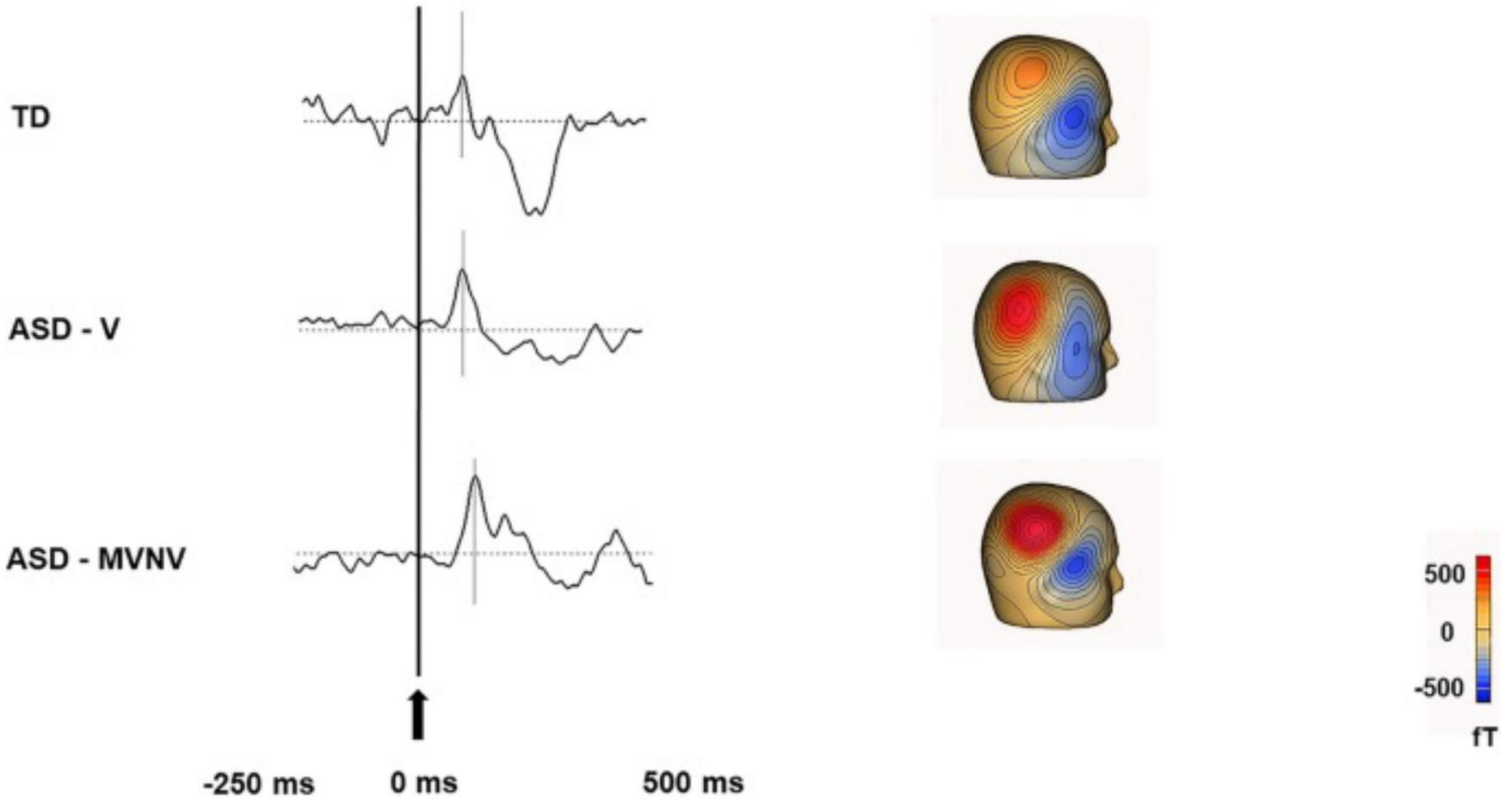
Delayed auditory event related field in ASD (adapted from Roberts TPL, Matsuzaki J, Blaskey L, et al. Delayed M50/M100 evoked response component latency in minimally verbal/nonverbal children who have autism spectrum disorder. Mol Autism. 2019;10:34. doi: 10.1186/s13229-019-0283-3; https://creativecommons.org/licenses/by/4.0^40^): The 3 waveforms show representative auditory evoked responses in a typically developing (TD) child (top), a verbal child with ASD who has no intellectual disability (ASD-V; middle) and minimally verbal or nonverbal child (MVNV) with ASD (bottom). The gray lines indicate the expected response at 50 ms (M50 peaks). For the representative TD child a response was observed around 71 ms, for the representative ASD-V child at 81 ms, and for the representative ASD-MVNV child at 98 ms. Abbreviations: ASD = autism spectrum disorder.

A significant body of literature has examined auditory processing in children with ASD, and its relation to language abilities^40^. These studies have generally shown that the primary auditory evoked field components (known as the M50 [∼50 ms latency] and M100 [∼100 ms latency]) are delayed in those with ASD^40^ (see Figure 3). These effects are seen from young children through to adults^41^ and are associated with language proficiency. Importantly, these MEG studies only involve children listening passively to auditory tones and therefore can, with training, usually be completed even in minimally verbal children^42^. Studies have further suggested that the slower auditory responses in ASD may be related to lower gamma‐aminobutyric acid (GABA) concentration (an inhibitory neurotransmitter) measured using magnetic resonance imaging, suggesting altered neurotransmission in ASD^43^.

There is also a model that dysregulated neural oscillations underlie the sensory atypicalities frequently reported in ASD; this has been assessed with auditory steady-state evoked fields, where reduced gamma power correlated with language impairment^44^^,^^45^. Other studies relate gamma band activity in the auditory cortex to GABA concentrations, showing atypical associations between the 2 metrics in ASD compared to typically developing (TD) youth^46^. When simple speech sounds are studied, receptive auditory processing was shown to be linked with speech articulation^47^, and abnormal brain dynamics have been shown to underlie speech production^48^.

Auditory processing difficulties have also been reported in ADHD. In fact, a substantial overlap between ADHD and central auditory processing disorder has been reported previously^49^, suggesting a close link between auditory processing difficulties and attentional problems. While only a few MEG studies have investigated auditory processing in children with ADHD, they have shown abnormal synchrony of the auditory evoked responses (M50) between left and right hemispheres^50–52^. For instance, one study investigated the volume of Heschl’s gyri and the auditory evoked responses in a cohort of children with and without musical training (*n* = 11; 7-9 years) and children with ADHD (*n* = 21)^51^. The authors found that children with ADHD had reduced volumes of Heschl’s gyri and decreased inter-hemispheric M50 synchrony, while children with musical training showed the opposite pattern. This finding of atypical hemispheric synchronization (ie, earlier M50 latency in the right compared to the left hemisphere) to a simple auditory syllable task was also shown to be associated with ADHD-like symptoms in 3-7-year-old children with ASD using the 151-channel KIT child-sized MEG system^52^. Thus, auditory processing differences may be a promising early indicator of ASD or ADHD-like symptoms, as well as a predictor of comorbidity.

Across these studies, the striking consistency of atypical auditory responses in those with ASD and ADHD strongly supports the presence of underlying neural abnormalities within the auditory system, which may contribute to language and attentional difficulties. These studies suggest that MEG is a key modality to derive putative biomarkers for a diagnosis of both neurodevelopmental disorders (see Roberts et al^53^ for an extensive review). However, measurement of such biomarkers in very young children (eg, to enable early diagnosis and possible intervention) remains challenging due to the limitations of current MEG systems.

### Face and emotional face processing

MEG studies have investigated the spatio-temporal signatures of evoked fields during face processing in children with ASD^54^^,^^55^. For example, Leung et al^54^ examined the implicit processing of happy and angry faces in children with ASD and TD peers (7-10 years). Compared to TD controls, children with ASD showed reduced source power to happy and angry faces in the left thalamus and right posterior cingulate gyrus, both key regions in processing visual and emotional stimuli, which may reflect difficulties in information processing that contribute to social impairment.

Atypical neural oscillatory responses to faces in the gamma frequency band have also been documented using MEG in children with ASD. These studies have shown absent or attenuated gamma responses in primary visual and other emotional face-processing areas^56^^,^^57^. For example, Wright and colleagues showed reduced induced gamma amplitude (30-80 Hz) in right lateral occipital areas and fusiform gyrus in ASD compared to TD children. Given that induced gamma oscillations are known to play an important role in early sensory feature binding^58^, these findings support a theory of disrupted processing of facial features in ASD. Further, a leading theory to explain the mechanism underlying the atypical gamma oscillations is the imbalance of cortical excitation and inhibition in neural systems^59^. As in the auditory system, this also suggests abnormalities in the GABA system, though more work is needed to confirm the specifics of this model.

**Figure 4.**
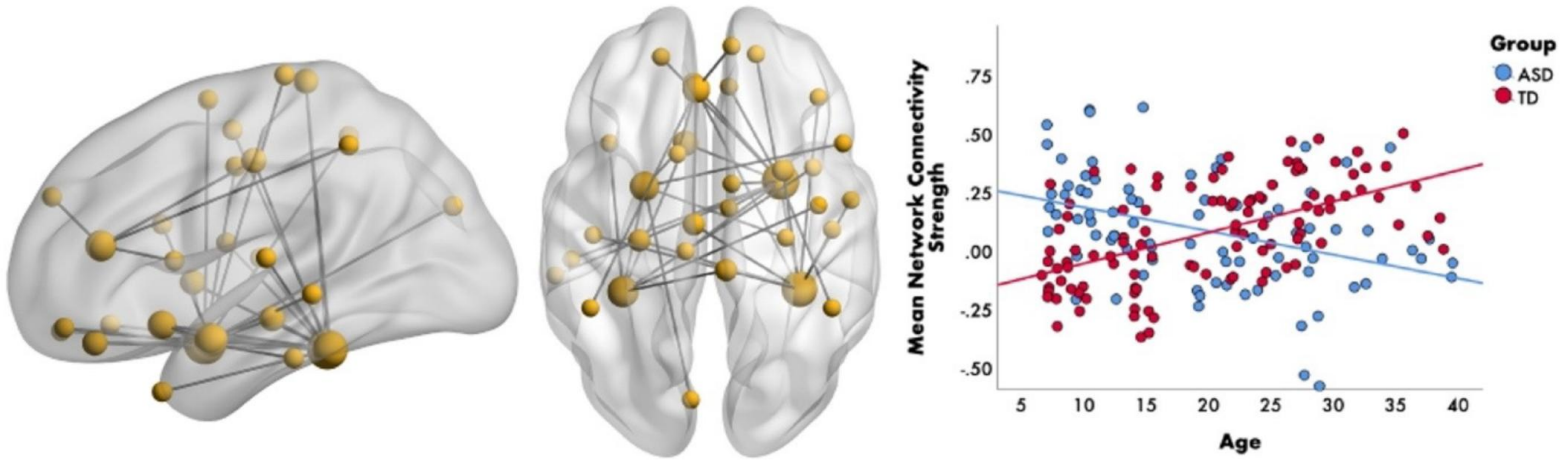
Age-related differences in gamma connectivity. Brain network (left-side) representing the significant group-by-age interaction to emotional faces in the gamma-band (30-55 Hz). The scatterplot (right-side) shows that connectivity strength within this gamma network increases with age in the TD group (in red) and decreases with age in the ASD group (in blue). From Safar K, Vandewouw MM, Taylor MJ. Atypical development of emotional face processing networks in autism spectrum disorder from childhood through to adulthood. Dev Cogn Neurosci. 2021;51:101003. License: CC BY-NC-ND 4.0.

Social cognitive function is mediated by temporal synchronization within brain-wide networks^60^ and, consequently, many studies of face processing in ASD have examined functional connectivity. MEG studies have demonstrated emotion- and frequency-specific alterations in networks associated with emotional face processing^61–64^. For example, Safar et al^63^ examined whole-brain functional connectivity of core face processing regions in ASD and TD children (7-10 years). Findings showed a network of increased alpha connectivity to happy faces in children with ASD, which correlated with higher autism severity scores. These findings suggest atypical communication among occipitotemporal areas (important for the early perception of emotional faces) along with orbitofrontal and limbic areas (involved in the evaluation of the emotional meaning of facial expressions). Another recent study in a large sample (*n *=* *190; 6-39 years), demonstrated that TD individuals showed age-related increases in gamma connectivity to emotional faces, while those with ASD demonstrated decreased connectivity with age (see Figure 4)^62^. These results suggest that emotion-processing networks undergo protracted maturation in TD and highlight the differences in the neurodevelopmental trajectory in ASD.

Although not a primary feature of ADHD, those with the condition demonstrate difficulties with emotional face processing^65^ and have a high degree of symptom overlap and comorbidity with those with ASD^66^. Despite this, there is a lack of MEG studies examining the neural underpinnings of emotional face processing in ADHD, as well as across both ADHD and ASD compared to a TD group. In a large study (*n* = 258), Safar et al^61^ found that children with ASD showed reduced whole-brain connectivity in the beta band, compared to TD children in response to faces, a further reduction in connectivity was seen in ADHD children compared to both ASD and TD, which was correlated with poorer scores of attention. These results suggest difficulties in the allocation of attention in ADHD. Further, in response to happy vs angry faces, ASD children displayed reduced gamma connectivity, while the opposite pattern was seen for the TD and ADHD children. However, a data-driven clustering approach which remained agnostic to the diagnostic labels identified 2 subgroups who differed in their functional networks: one which was predominantly TD individuals, and the other which was predominantly ASD and ADHD. The lack of distinct ASD and ADHD subgroups suggests the presence of shared functional connectivity across the 2 neurodevelopmental disorders, aligning with a growing body of work showing the overlap in both biology and phenotype in these 2 conditions (e.g., Vandewouw and colleagues^67^).

In summary, these findings demonstrate reduced gamma responses and altered connectivity networks in the beta and gamma frequency bands in ASD and ADHD, known to be important for attention and the perception of faces^68^, as well as the similarity of the neural substrates across these neurodevelopmental disorders, which may relate to overlapping symptomology. This demonstrates the utility of emotional face paradigms and measurements of functional connectivity using MEG for characterizing differences in neural processing between clinical groups and TD children.

### Task-free MEG studies in ASD and ADHD

While task-based experimental paradigms can offer valuable insights into specific functions, they also place a heavier cognitive burden on participants. Task-free paradigms (typically termed resting-state) are useful alternatives, as they are generally easier to complete in younger children and clinical populations allowing for more useable data to be collected. Resting-state paradigms vary but most involve participants being presented with either a fixation cross, passively viewing a visual stimulus, or resting with their eyes open or closed. While most resting-state studies in ASD and ADHD have focused on functional connectivity, some have investigated group differences in regional oscillatory power or “peak alpha frequency” (PAF) (ie, the frequency at which alpha oscillations demonstrate maximum power).

Resting-state alpha power and PAF are affected in children with ASD. One study found that children with ASD showed region-specific increases in power across multiple frequency bands compared to TD children (6-17 years)^69^. Importantly, within the ASD group, increased alpha power in temporal and parietal regions correlated with greater social impairment. Previous EEG and MEG studies also suggest that PAF may be a sensitive marker of cognitive development^2^^,^^70^. It may also serve as a useful clinical marker for ASD, as it shows high heritability and test-retest stability^71^^,^^72^. Supporting this, several MEG studies have shown age-related increases in alpha (7-13 Hz) in TD but not ASD children, suggesting atypical maturation of the PAF^73^^,^^74^. Edgar and colleagues investigated PAF in a large cohort of males with ASD (*n* = 183; 6-17 years) and TD children (*n* = 121). They found that PAF was higher in the ASD group, but only in the younger age range (6-10 years), which may reflect accelerated early maturation of resting-state alpha oscillations^73^. In line with this, another study found higher PAF in a group of 6-9-year-old males with ASD (*n* = 45) compared to TD (*n* = 47) children. They also found a significant positive association between PAF and non-verbal IQ in the TD group^74^. While both these studies reported higher PAF in ASD compared to TD children, this appears most pronounced at younger ages (<10 years)^73^, suggesting it may be a more sensitive marker of ASD in early development. Further research should determine if PAF is a useful clinical marker for ASD and across what age range. In addition, as most of the aforementioned studies were done in males^73^^,^^74^, future work should prioritize the recruitment of females with ASD to explore possible sex differences.

MEG studies have allowed for the delineation of frequency-specific resting-state networks in children with ASD. Using a child-sized 151-channel KIT system, Kikuchi et al^75^ investigated resting-state connectivity at the sensor level in ASD (*n* = 35) and TD (*n* = 35) children (3-7 years). The authors found increased right-lateralized gamma connectivity between parietotemporal areas in ASD^75^. Gamma hyper-connectivity has also been reported in older children and adolescents (12-15 years) with ASD in orbitofrontal, subcortical, and temporal regions, which are implicated in social cognition^76^. Another MEG study spanning a wider age range (6-21 years) investigated graph theory metrics reflecting global integration and local segregation across networks^77^. The authors found that group differences were most prominent in the gamma band, suggesting increased local and global efficiency in ASD compared to TD peers. Importantly, within the ASD group, increased local network connectivity in gamma was associated with symptom severity^77^. These findings are consistent with a theory of local over-connectivity in ASD, as gamma oscillations are thought to mediate synchronization in local circuits^78^.

The resting-state MEG literature in youth with ADHD is more limited. To our knowledge, 5 studies have been conducted on children with ADHD, and most with small sample sizes of 14 or fewer participants^79–83^. For instance, one study examined sensor-level connectivity in 13 children with ADHD and 14 TD children (8-13 years)^80^. Their results revealed hyper-connectivity in delta, beta, and gamma bands within anterior and central brain regions in children with ADHD compared to TD children, suggesting altered maturation of frontal cortices in ADHD. Consistent with this, another study found that brain complexity (measured using Lempel-Ziv complexity scores), particularly in anterior brain regions, was decreased in children with ADHD (*n* = 14) compared to TD peers (*n* = 14), further supporting the theory of delayed cortical maturation in ADHD^84^. Interestingly, the authors also found an increase in complexity scores with age in TD children, while the ADHD group showed a trend (non-significant) towards decreased complexity scores with age, suggesting diverging developmental trajectories. A recent study employing both EEG and MEG found alterations in thalamo-cortical connectivity in children with ADHD compared to TD children in alpha, beta, and gamma frequency bands^83^. Using machine-learning techniques, the authors were able to differentiate between ADHD and TD groups with a high degree of accuracy, 98% and 97% accuracy for EEG and MEG, respectively. Despite the limited resting-state studies in ADHD using MEG, the existing literature provides important insights into the connectivity profiles of children with ADHD, highlighting the vulnerability of frontal regions. Additional research in larger datasets is warranted to better understand the neurophysiological basis of ADHD, particularly the frequency bands and networks affected.

A recent development in the resting-state literature is the use of movie-like paradigms to improve compliance during scans. As previously mentioned, head motion is a significant challenge in paediatric neuroimaging and fMRI studies have shown that head motion is increased during resting state compared to tasks^85^. To address this, Vanderwal and colleagues developed a non-verbal movie paradigm called *Inscapes* that consists of slowly moving abstract shapes intended to sustain engagement. A recent MEG study showed that head motion was reduced during *Inscapes* compared to fixation cross in TD, ASD, and ADHD youth (5-19 years)^86^. Importantly, results showed the differences in oscillatory power and functional connectivity in *Inscapes* compared to fixation cross were similar in TD, ASD, and ADHD groups, making it an appropriate paradigm for investigating resting-state activity in children with neurodevelopmental disorders. Thus, *Inscapes* is a promising paradigm for use in young children and clinical populations, to improve compliance and hence data quality^86^^,^^87^.

Taken together, these findings highlight the oscillatory brain activity and connectivity differences during task-free resting-state, particularly in alpha and gamma frequency bands, in children with ASD and ADHD. While there are some conflicting findings, there seems to be a general consensus implicating altered gamma connectivity during rest, extending the face processing literature showing atypical recruitment of gamma oscillations in ASD. Given the relative ease of acquiring resting-state data, we believe that MEG is a valuable tool for assessing developing changes in intrinsic brain function in both typical and atypical development. However, studies in children with ADHD with larger sample sizes, as well as transdiagnostic approaches to investigate shared patterns of functional connectivity, are warranted to confirm the neurophysiological mechanisms underlying social and cognitive processes.

## The future of paediatric MEG

**Figure 5.**
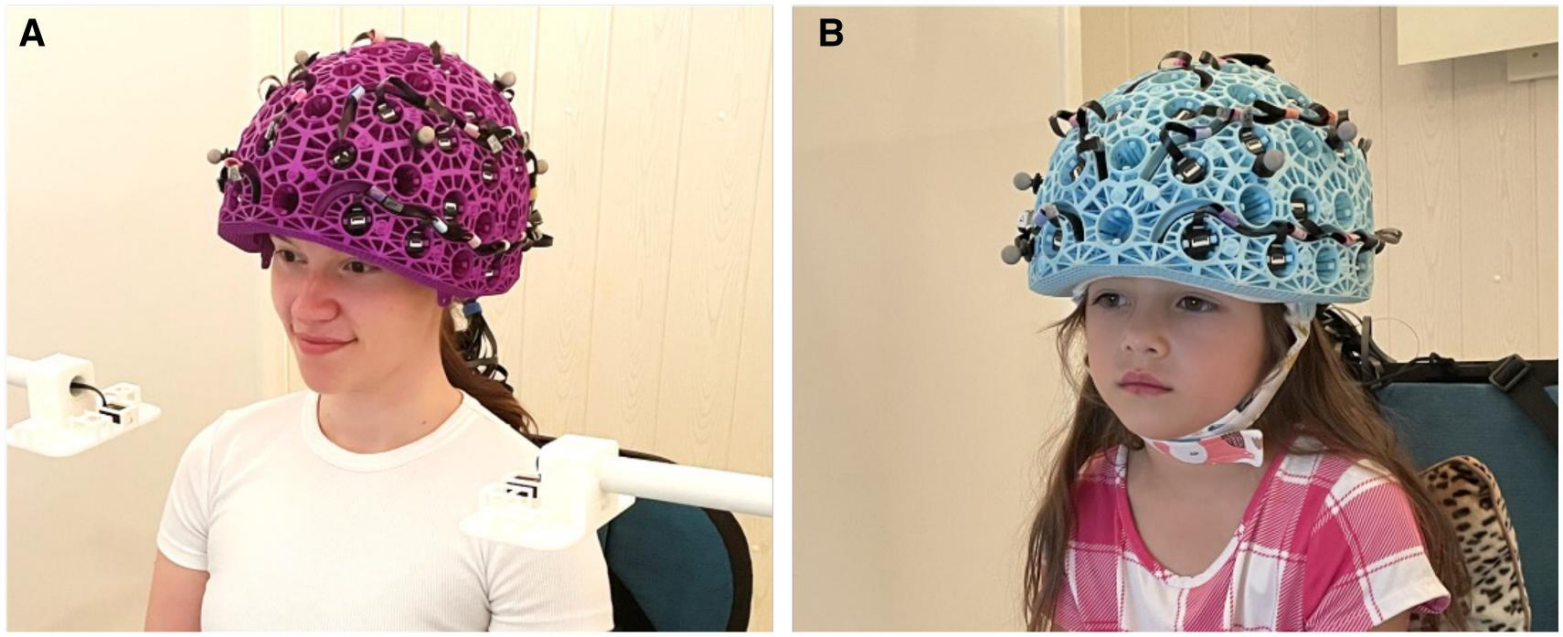
OPM-MEG system with (A) an adult helmet and (B) a child helmet.

Despite the significant promise offered by MEG, its use in neurodevelopment has been limited by the difficulties in acquiring MEG data from very young children. This problem may be addressed by recent changes in MEG instrumentation. OPMs manipulate the quantum mechanical properties of atoms using laser light, resulting in an atomic ensemble that is highly sensitive to small changes in magnetic field (for a review, see Schofield et al^88^). OPMs are small and lightweight and can be mounted inside wearable helmets (see Figure 5) accommodating (in principle) any head size. Crucially, they do not require cryogenic cooling and can be operated close to the scalp, providing higher signal amplitude (due to proximity) and spatial resolution. Assuming background fields are controlled, subjects can move during a scan, providing a more comfortable naturalistic scanning environment better suited to children. The first use of a wearable OPM-MEG array was described in 2018^89^ and has since been validated across a number of studies, including motor^90^, cognitive^91^^,^^92^, and visual^93^, successfully measuring evoked fields^90^^,^^93^, oscillations^91^^,^^92^^,^^94^^,^^95^, and connectivity^15^.

To date, most OPM-MEG studies have been based on adult populations, but the technology has enormous potential as a tool for paediatric neuroimaging. One study using an early prototype OPM-MEG system, comprising a small focal array of sensors, measured signals during a sensory task from a 2- and a 5-year-old child, successfully detecting beta modulation in both children^94^. A later technical study used triaxial sensors (ie, OPMs which measure the magnetic field in 3 orientations) to characterize a sensory response in a 6-year-old child^95^. This also showed the advantage of multi-axis field measurement to prevent inhomogeneous coverage of the infant’s brain when OPM sensors get very close to the head. A recent study in paediatric epilepsy compared the ability of cryogenic MEG and OPM-MEG to detect and localize IEDs in 5 school-aged children with epilepsy^96^. They found higher SNR, higher amplitude, and similar localization with OPM-MEG compared to cryogenic MEG^96^. An exciting study from the same group demonstrated, in a single 10-year-old child, the ability to detect MEG signals during a seizure^97^. A study by Hillebrand et al^98^ successfully measured ictal and inter-ictal activity in children (age 10-12 years) and adults using a 12-channel OPM-MEG system, again comparing results to those derived from conventional MEG and showing a high degree of consistency. This is a limited number of studies (reflecting the age of the technology), yet they already begun to demonstrate significant promise of OPM-MEG in measuring electrophysiological signals in the very young.

Compared to EEG, OPM-MEG (and MEG in general) is less sensitive to muscle artefacts caused by movement^99^, especially in the gamma range, which (as demonstrated above) is critical for the study of neurodevelopment. The ability to readily adapt to different head sizes in the early childhood period ostensibly removes the inherent confound of SNR in neurodevelopment that is associated with conventional MEG, providing the ability to conduct both longitudinal and cross-sectional studies. A more friendly scanning environment (where patients can move) is likely to increase compliance and consequently improve data retention rates. Perhaps most importantly, it will enable the acquisition of MEG data from much younger patients and more disabled patients, meaning that it will be possible to observe the myriad MEG markers (described above) that accompany ASD and ADHD, before and after symptoms emerge in early childhood. It should be noted that the technology remains in its early stages, an established design for a paediatric OPM-MEG system has not yet been reached, and there remain technical issues to be solved. Nevertheless, OPM-MEG offers a new and exciting MEG platform to investigate neurodevelopmental disorders.

## Conclusion

MEG provides an excellent platform to study the trajectories of brain function in both typical and atypical development, benefitting from the combination of outstanding temporal resolution and high spatial resolution. While currently only clinically approved for epilepsy, MEG has the potential for wider clinical relevance, including for ASD and ADHD. While conventional MEG technology remains expensive and difficult to deploy, particularly in younger subjects, new developments in MEG technology are likely to inspire an increase in both research and clinical uptake, particularly in neurodevelopment and the disorders that affect it.
